# Laryngeal and Voice Disorders in Patients with Pulmonary Tuberculosis

**DOI:** 10.22038/ijorl.2020.47194.2550

**Published:** 2021-03

**Authors:** Gamal Youssef, Bassam-Hasan Mahboub, Safinaz-Nagib Azab

**Affiliations:** 1 *Department of Otolaryngology, Faculty of Medicine, Alexandria University, Egypt and Consultant Phoniatrics, ENT Department, Dubai Hospital, DHA, Dubai, UAE.*; 2 *Consultant and Head of Pulmonology Unit, Dubai Hospital, DHA, Dubai, UAE.*; 3 *Department of Otolaryngology, Faculty of Medicine, Bani-Suef University, Egypt.*

**Keywords:** Dysphonia, Laryngeal Tuberculosis, Laryngostroboscopy, Pulmonary TB

## Abstract

**Introduction:**

Laryngeal tuberculosis (LTB) is the most frequent granulomatous disease of the larynx. The aim of the present work was to study the laryngostroboscopic features and voice quality of patients with laryngeal TB secondary to pulmonary TB.

**Materials and Methods:**

Participants were 35 patients diagnosed as having pulmonary TB and dysphonia. All patients had a complete history, clinical and laboratory workup. Patients were assessed using a protocol of voice assessment which included Auditory-perceptual analysis of voice, voice analysis using the Multidimensional Voice Profile (MDVP), and laryngostroboscopy.

**Results:**

The participants were 24 males and 11 females and their mean age was 43.7 years. The voice acoustic analysis revealed a significant difference from normal in jitter percent, shimmer percent, and harmonic to noise (H/N) ratio. Laryngeal gross lesions were found in 11 patients while the other 24 patients had normal laryngoscopic findings with nonspecific stroboscopic changes as reduced mucosal waves and mild glottic gap. Diffuse lesion of the whole vocal folds was found in 5 patients and anterior predilection in 4 patients. The type of lesions were granulomatous lesions in 7 patients and non-specific inflammatory mild exophytic lesions in 4 patients.

**Conclusions:**

Voice disorders in pulmonary TB include disturbance in the mechanism of voice production with or without detectable laryngeal lesion. Videostroboscopy has the advantage of showing the extension of laryngeal involvement, vocal folds vibrations, and mucosal waves.

## Introduction

Tuberculosis (TB) is a chronic granulomatous disease caused by Mycobacterium Bacilli. Laryngeal tuberculosis (LTB) is the most frequent granulomatous disease of the larynx. Most cases of LTB demonstrated lung implication; however, LTB can be found without pulmonary involvement (1). 

Any laryngeal anatomic site can be affected by TB, with great variability in clinical and endoscopic pictures (2). Studies revealed that the incidence of laryngeal involvement among pulmonary TB patients varies from 0.08 to 5.1% (1-3). However, the actual incidence is higher and difficult to be determined due to a lack of routine systemic laryngeal evaluation. In addition, most of the young physicians overlook TB in the differential diagnosis of laryngeal disorders, resulting in wrong diagnosis and treatment resulting in the spread of infection, which can become a public health problem (4).

In Middle East countries, there is a decline in the incidence of tuberculosis due to improvement in antituberculosis drugs, and in the standard of living. However, the incidence of tuberculosis has been increased as a result of the presence of HIV and immigrants from countries of high TB prevalence (5-6). 

The TB infection has initial exudation in the lamina propria followed by infiltration of round cells and finally heals with fibrotic changes in the lamina propria affecting the vibrations of the vocal folds and causing permanent changes in the voice quality. Stroboscopy can diagnose abnormalities of vibratory patterns, asymmetry, reduction of amplitude and mucosal wave (7-9). 

Voice changes and acoustic analysis of patients with pulmonary TB have not been investigated in previous literature. In addition, the use of laryngostroboscopy in the assessment of LTB has not been sufficiently studied in the literature. 

The aim of the present work was to study the laryngostroboscopic features and voice quality of patients with laryngeal TB secondary to pulmonary TB in order to reach an accurate diagnosis and appropriate treatment of voice disorders in patients with pulmonary TB.

## Materials and Methods

Participants involved in the study were 35 patients diagnosed as having pulmonary TB and referred from pulmonology Unit to voice and ENT clinic at Dubai hospital for investigation of persistent dysphonia between June 2018 and December 2019. All patients had a complete history, clinical and laboratory workup. According to American Thoracic Society, active pulmonary tuberculosis was defined as a positive smear and/or culture for acid-fast bacilli, clinical symptoms, and positive chest X-ray (CXR) films. The inactive TB was based on a positive tuberculin skin test, the absence of clinical, bacteriological, or radiographic evidence of active disease, and CXR findings coherent with past TB (10). 

Patients were assessed using a protocol of voice assessment which included Auditory-perceptual analysis of voice using the modified GRBAS scale (Grade, Roughness, Breathiness, Asthenia, and Strain) and it was performed by expert phoniatricians.

The voice analysis was done using the Multidimensional Voice Profile (MDVP) in Kay CSL 450. Participants were asked to phonate a soft vowel with a comfortable pitch at distance 30 cm from the microphone and the room ambient noise level was 30 decibels. Data collected from MDVP includes the fundamental frequency, jitter percent, shimmer percent, and Harmonic to noise (H/N) ratio (9,11). Laryngostroboscopy was done using the video-laryngostroboscope model (STORZ AIDA WD 200) using a rigid telescope passed orally or flexible nasopharyngoscopy passed nasally to assess the presence and location of the mucosal lesions. The parameters obtained from stroboscopy were the extent, regularity and velocity of the traveling wave, mucosal wave pattern, and glottic closure pattern (9). According to laryngoscopic findings, patients either have a detectable laryngeal lesion or no lesion has been detected during the laryngoscopic examination. 

The lesions are described regarding their nature (granulomatous, polypoid, flat exophytic, nonspecific inflammatory or ulcerative), location (vocal fold, ventricular fold, aryepiglottic fold, epiglottis or others), laterality (Right, left or bilateral) and predominance (anterior or posterior) (2).Statistical analyses were performed with SPSS 20.0 software (SPSS Inc, Chicago, IL). Descriptive statistics were used to measures qualitative variables using frequency and percentage. Numerical variables were described using mean and standard deviation. One sample t-test was used to study significant differences between acoustic measures mean in TB patients and normal means, and a P<0.05 was considered significant.The Institutional Review Board at Dubai Health Authority approved the protocol of this study and written informed consent was obtained from the patients.

## Results

This study is a descriptive cross-sectional study and included 35 patients with dysphonia and confirmed pulmonary TB under treatment protocol in the pulmonology unit. All patients have pulmonary TB confirmed by chest x-ray and sputum culture and smear. Table 1 shows the clinical and demographic data of the patients. The participants were 24 males and 11 females and their age mean was 43.7 years and ranged from 26 to 64 years. The dysphonia started one to three months after pulmonary symptoms in most cases with a range from 15 days to 8 months. 

**Table 1:** demographic and clinical data of studied patients

**Variables**	**Group**	**Frequency**	**Percentage**
gender	Male	24	69%
	female	11	31%
dysphonia		35	100%
Weight loss		13	37%
dysphagia		7	20%
fever		17	49%
Smoking		13	37%
Pulmonary TB	active	20	57%
	inactive	15	43%
Continuous variables			
variable	Mean	SD	Range
age	43.7	±21.7	26-65
Duration of chest symptoms (Months)	Mean 7.2	SD 5.07	Range1-23
Duration of dysphonia	2.6	1.4	0.5-6
Duration of treatment	3.2	2.1	.25-8

The results of APA showed that all patients had dysphonia of mild to a moderate degree and mixed quality. The voice quality was strained-asthenic in 17 patients, rough-in 15 patients, and breathy in 3 patients.

The voice acoustic analysis using MDVP revealed a significant difference from normal in jitter percent, shimmer percent, and harmonic to noise (H/N) ratio as demonstrated in table 2.

**Table 2:** Results of the acoustic analysis in the studied patients

**Variable**	**Mean**	**SD**	**Normal mean**	**T value**	**Sign ( 2 tailed)**
Fundamental frequency (HZ)	109.12	28.4	125.59	-1.63	.146
Jitter percent	1.1137	.143	.449	8.06	0.015
Shimmer percent	3.87	.964	0.159	6.67	0.022
Noise to Harmonic Ratio NHR	.148	.011	.143	.801	0.506

The Laryngoscopic findings are listed in table 3 which revealed that laryngeal gross lesions were found in 11 patients while the other 24 patients had normal laryngoscopic findings with nonspecific stroboscopic changes. Diffuse lesion of the whole vocal folds was found in 5 patients, anterior predilection was found in 4 patients, while the posterior lesion was found in 2 patients. 6 patients had bilateral involvements and additional sites involved were aryepiglottic folds in 2 patients, and ventricular folds in 3 patients as well as vocal folds involvement. No epiglottic lesions were observed in our patients. Stroboscopic examination revealed reduced mucosal waves over the lesion in 7 patients, asymmetrical vocal folds vibration in 6 patients, and decreased amplitude in 9 patients. A small to moderate glottic gap was found in 5 patients and a fusiform glottic gap detected in 4 patients. Stroboscopic examination in patients without detectable lesion in the larynx was normal in 8 patients, reduced amplitude in 12 patients, a small glottic gap in 9 patients and supraglottic hyperfunction in 12 patients. The stroboscopic examination was not possible in 7 patients due to severe gagging.

The type of lesions detected were granulomatous tumor-like lesions in 7 patients and non-specific inflammatory mild exophytic lesions in 4 patients. 

No ulcerative or polypoid lesions were noted in our patients. 

**Table 3:** Laryngoscope findings of studied patients

**Site of the lesion**	**Whole Vocal fold**	**Anterior predominance**	**Posterior predominance**	**Aryepiglottic ** **fold**	**Ventricular folds**
	5	4	2	2	3
Side of the lesion	Right vocal fold	Left VF	Bilateral	Multiple sites	
	3	2	6	5	
Type of the laryngeal lesion	Detectable gross lesion			No detectable gross lesion	
	11			24	
	granulomatous	Nonspecific exophytic	ulcerative	Congested vocal folds	Normal vocal folds
	7	4	0	14	10
Stroboscope	Reduced MW	Decrease amplitude	Glottic gap	Supraglottic hyperfunction	Not done
	16	11	18	12	7

## Discussion

Tuberculosis (TB) is a major cause of illness, one of the highest 10 causes of death globally and the top cause of mortality from a single infective cause (rating above HIV/AIDS). Globally, an estimated 10.0 million people had TB in 2018, a figure that has been quite stable in the latest years (6). The incidence of LTB in pulmonary tuberculosis has been markedly declined, to less than 1% of tuberculosis cases due to the development of ant-tuberculous drugs and improvements in public health (11). The sex distribution of subjects in the present study shows a male predominance with a mean age of 43 years, consistent with the previous studies results (12-14).

There are two different theories that explain laryngeal TB affection. The most-reported theory is the bronchogenic theory. The infection reaches the larynx via direct contamination by the lung secretion rich in bacillus. So, the laryngeal settlement is secondary to lung disease (6-8). According to that theory, the posterior larynx, where the infected sputum was accumulated, is the most commonly affected area in secondary LTB (13-14). The second less common theory explaining laryngeal infection is the hematogenic theory. The bacillus reaches via the blood or lymphatics and the lesions are more common in the anterior larynx (3). The site of TB laryngeal lesion in the present study was in the vocal folds in all cases with a predilection for the anterior part of vocal folds, along with more prevalence of whole vocal folds affection. These laryngeal site predilection in the present study reflect changing styles of laryngeal TB as documented in previous studies (12-17). Lim finds anterior preference of TBL due to the spread of laryngeal tuberculosis via the hematogenous or lymphatic spread, which is going up in most cases at present, more than bronchogenic direct spread (17). In addition, the anterior predilection can be attributed to ambulatory treatment régimes which decrease sputum accumulation in the posterior larynx (4). 

The type of lesion predominate in our study was granulomatous in 64% and nonspecific exophytic in 36% of patients with positive lesions. However, no polypoid or ulcerative lesion was noted in our patients. Lim in his study of 60 cases of laryngeal TB categorizes laryngeal lesion into 4 types; granulomatous, polypoid, ulcerative, and nonspecific hypertrophic lesion. They assumed that patients with active pulmonary TB show more granulomatous lesion while the patient with inactive pulmonary TB show more nonspecific lesions (17). Several studies documented changes in the trend of laryngeal Tb in the last decade (12-17).Our study revealed that 68% of the patients with dysphonia and pulmonary TB showed no detectable gross lesion in laryngoscopy. However, the laryngostroboscopic findings were reduced mucosal waves, a small glottic gap in phonation, and supraglottic hyperfunction. Previous studies found a complete resolution of the laryngeal lesion within 2 to 9 months after starting antituberculosis treatment (18). Our patients were already on a treatment protocol that may change the nature of the disease. The mean duration of treatment before referral for laryngeal evaluation was 3 months and ranged from 1 week to 8 months so, the laryngeal findings in our study can show all stages of the disease from initial lesion to recovery stage according to the duration of treatment. Follow up endoscopy done for 5 patients after 2 months of initial assessment and revealed a reduction in the size of the lesion in 2 subjects, and complete disappearance in 3 patients. The treatment itself may cure the laryngeal lesion but sequel of fibrosis may impact voice negatively.

Another explanation of dysphonia, without gross laryngeal lesion, is functional elements of dysphonia with Voice misuse due to cough and smoking. Cough and smoking may play a role in voice and laryngeal changes due to vocal folds trauma and irritation that results in vocal folds congestion, mucosal changes, and hyperfunction voice disorders. In addition, Pulmonary TB itself reduces pulmonary functions that affect aerodynamic powers at glottis that disturb mucosal waves and voice quality. Studies on patients with COPD, without any specific laryngeal infection, found abnormal mucosal waves pattern in addition to vocal fold erythema or irregularities (19). 

The laryngostroboscopic findings in laryngeal lesions, in the present study, were in agreement with previous studies as videostroboscopy can better display the spread of involvement in the vocal fold layers. Findings on videostroboscopy include decreased mucosal wave, glottic waste, asymmetric vocal folds vibration and vocal fold scarring disproportionate to the visible lesion (20). Lucena approved that alteration of vocal folds vibration begins throughout the active phase of the disease and is continued due to the scarring process or by functional adjustment mechanisms settled during the functional voice limitation phase (21). 

The laryngeal TB lesions, such as ulceration, granuloma, and fibrosis can hinder the voice production process. The involvement of the lamina propria can change the vocal folds flexibility and, cause dysphonia which is the main symptom present in most of the cases (4). These voice changes have been documented by alteration in acoustic parameters like jitter, shimmer, and harmonic to noise ratio (22).

In the present study, the histological evaluation of laryngeal lesion biopsy was not done, but a combination of positive smear and/or culture, clinical symptoms and positive chest X-ray and prompt response to antituberculosis treatment (ATT) confirmed the diagnosis of laryngeal TB. These criteria for the diagnosis of laryngeal TB used frequently in previous studies without performing a histopathological examination. Some researchers assumed that response to ATT is an important diagnostic criterion for laryngeal tuberculosis. They concluded that avoiding biopsy is safe and logical, as giving general anesthesia to an active pulmonary TB case is not always possible, due to anesthesia worries, drug interactions, and infectivity matters (12,23,24). To our knowledge, no previous researches studied functional voice disorders in Pulmonary TB. The study raises the importance of proper management and early detection of voice and laryngeal disorders in patients with pulmonary TB. Clinicians managing patients with pulmonary TB should bear in mind that indicators of laryngeal involvement may be minor, and laryngostroboscopy should always be performed when suspecting laryngeal involvement, with the purpose of, early detection and management of voice and laryngeal disorders in these patients (25). 

## Conclusion

Dysphonia is the most frequent symptom of LTB and a common problem of pulmonary tuberculosis. Voice disorders in pulmonary TB include disturbance in the mechanism of voice production with or without detectable laryngeal lesion. Videostroboscopy has the advantage of showing the extension of laryngeal involvement, the nature of vocal folds vibrations, and mucosal waves. However, the stroboscopic pictures are variable depending on the type and nature of the lesion. The otolaryngologist must sustain high suspicion and awareness in dealing with patients with pulmonary TB and dysphonia. Functional elements of voice disorders should be assessed properly in a patient with pulmonary TB, in the meantime, voice therapy could help in the prevention of compensatory voice disorders and improve the quality of voice. 
